# Parasitological, Hematological and Biochemical Characteristics of a Model of Hyper-microfilariaemic Loiasis (*Loa loa*) in the Baboon (*Papio anubis*)

**DOI:** 10.1371/journal.pntd.0004202

**Published:** 2015-11-10

**Authors:** Samuel Wanji, Ebanga-Echi Eyong, Nicholas Tendongfor, Che Ngwa, Elive Esuka, Arnaud Kengne-Ouafo, Fabrice Datchoua-Poutcheu, Peter Enyong, Adrian Hopkins, Charles D. Mackenzie

**Affiliations:** 1 Parasites and Vectors Research Unit, Department of Microbiology and Parasitology, Faculty of Science, University of Buea, South West Region, Cameroon; 2 Research Foundation for Tropical Diseases and Environment (REFOTDE), South West Region, Cameroon; 3 Department of Biological Sciences, Faculty of Science, University of Bamenda, North West Region, Cameroon; 4 Department of Zoology and Animal Physiology, Faculty of Science, University of Buea, South West Region, Cameroon; 5 Mectizan Donation Programme, Decatur, Georgia, United States of America; 6 Department of Pathobiology and Diagnostic Investigation, Michigan State University, East Lansing, Michigan, United States of America; University of Liverpool, UNITED KINGDOM

## Abstract

**Background:**

Loiasis, a filarial infection caused by *Loa loa* usually thought to cause relatively minor morbidity, can cause serious and often fatal reactions in patients carrying very high levels of circulating *Loa loa* microfilariae (mf) following administration of microfilaricidal drugs. An experimental model of this condition would greatly aid the definition of the optimal management of this important clinical presentation.

**Methodology/Principle Findings:**

Fifteen baboons (*Papio anubis*) were infected with 600 infective larvae (L3) isolated from *Chrysops* vector flies. Animals were observed for any clinical changes; blood samples were collected every 1–2 months for 22 months, and analysed for parasitological, hematological and biochemical profiles using standard techniques. All animals became patent but remained clinically normal throughout the study. The parasitological pre-patent period was between 4–8 months, with a majority (60%) of animals becoming patent by 5 months post infection (MPI); all animals were patent by 8 MPI. Microfilarial loads increased steadily in all animals and reached a peak at 18 MPI. By 10 MPI >70% of animals had mf >8,000 mf/mL, and at 18 MPI >70% of animals had mf >30,000mf/mL with 50% of these animals with mf >50,000mf/mL. Absolute eosinophil, creatinine, Ca^2+^ and K^+^ levels were generally above normal values (NV). Positive associations were seen between microfilariaemia and eosinophilia, Hb, Ca^2+^, and gamma-GT values, whilst significant negative associations were seen between microfilariaemia and potassium, glucose and mononuclear leukocyte levels.

**Conclusions:**

Infection of splenectomised baboons with *L*. *loa* can induce levels of circulating microfilariae, and corresponding haematological profiles, which parallel those seen in those humans in danger of the severe post-microfilariacide clinical responses. Utilization of this experimental model could contribute to the improved management of the loiasis related adverse responses in humans.

## Author Summary

Loiasis is a filarial infection of humans that, in addition to causing severe direct clinical effects, is of concern to the global community’s efforts to eliminate the important filarial diseases, onchocerciasis and lymphatic filariasis, through causing interruption to mass drug distribution activities. Hyper-microfilariaemia has been seen to be the characteristic parameter in patients suffering from post ivermectin encephalopathy, a condition which sometimes leads to death. Understanding and developing appropriate approaches to the treatment and prevention of these severe adverse reactions has been difficult due to the lack of suitable models. As primates can be infected with human *L*. *loa*, and can develop hyper-microfilariaemia, it is likely that they therefore can serve as suitable models for the investigation of this syndrome in humans. This current study shows that following splenectomy the circulating microfilarial loads are similar to those seen in humans, and that the clinical pathology profile following infection also appears to be similar. The consistent ability to induce microfilariae levels of above 30,000 mf/ml in more than 70% of the tested animals suggests that this is indeed a practical model for investigating the adverse events occurring in hyper-loiasis.

## Introduction

*Loa loa* is a parasitic filarial nematode of humans, and a member the super family Filariodea which includes infections that are targeted for elimination, such as lymphatic filariasis (*Wuchereria bancrofti*, *Brugia sp)*, and onchocerciasis *(Onchocerca volvulus)*. *L*. *loa* is found in the tropics [1] restricted to the rainforest and forest fringes of West and Central Africa [2] and causing a relatively well tolerated medical condition known as loiasis. The geographic distribution of this infection is limited by the presence of the two biting tabaniid vectors, *Chrysops silicea* and *C*. *dimidiata*, which generally prefer rainforest-like environment; however, recently the disease has been described in the Guinean savannah [3]. It is estimated that some 14.4 million people live in high risk areas where the prevalence of loiasis (i.e. a history of “eye worm”) is greater than 40%, with 15.2 million in intermediate risk areas where the estimated eye worm prevalence is between 20 and 40% [4].

This disease has been recognized as one of public health importance, not so much because of its own clinical manifestations, but because of its negative impact on the control of onchocerciasis and lymphatic filariasis in areas of co-endemicity. There have been increasing reports in the past decade of serious adverse events (SAE) following the administration of ivermectin for these two major filariae, onchocerciasis and lymphatic filariasis, in *L*.*loa* endemic areas. These SAE are characterized by a severely disabling and potentially fatal encephalopathy. Evidence exists that these SAE appear to correlate with high loads of *L*.*loa* microfilariaemia (>30,000 mf/mL) [5–8].

Despite advances in defining the epidemiological aspects of these *L*.*loa* associated SAE, their pathogenesis and treatment still remains obscure. It is not known if the genesis of the encephalopathy is associated with the increased presence of *L*.*loa* in the brain tissue, although a vasculopathy associated with the presence of microfilariae has been proposed as a possible aetiology [9,10]. To be able to properly manage these cases, it is necessary to better understand the mechanism of pathogenesis of the post-ivermectin events in these heavily infected individuals. Very limited progress has been made in research on the pathogenesis of encephalopathy, due in most part to the lack of material from human cases, as well as a lack of any useful animal models to investigate the etiology and test new potential clinical management procedures.

Although the natural hosts of *L*. *loa* are humans, the entire life cycle of *L*. *loa* can be maintained experimentally. *L*. *loa* readily infects mandrills (*Mandrillus leucophaeus)* [11], baboons (*Papio anubis)* [12], and patas monkeys (*Erythrocebus patas)* [12]. Non-human primates (NHPs) are therefore considered the best models for the much needed investigation in human loiasis, especially as suitable *in vitro* models for loiasis have not yet been developed, and indeed such artificial models are not easily extrapolated to humans [13–15]. Although the mandrill is an excellent experimental host, there are ethical concerns with using this now protected animal for research, and it is no longer used in biomedicine. The use of *Patas* monkeys also is limited as the parasite does not behave in the same way like it does in the more human-like drill [12].

The baboon (P. *anubis)* however, has potential as an experimental model to study the mechanisms behind the SAEs that develop in *L*. *loa* infected people as in this animal the parasite here behaves essentially in the same way as it does in the drill [12], and therefore is comparable with the situation in humans. Secondly, the use of baboons in biomedical research is accepted by the International Union for Conservation of Nature-IUCN [16]. In the simian host, the spleen usually becomes enlarged and granulomatous in filarial infections as it is the site of destruction of a large proportion of circulating *Loa* microfilariae [17,18]. If the spleen is removed very high levels of circulating microfilariae develop in the blood (>50,000 mf/mL), levels that are similar to those found in patients developing the post-treatment *Loa-*associated encephalopathy. Thus the baboon, with its remarkable similarities to humans in many anatomical and physiological parameters [19, 20, 21], appears to likely be a suitable model for studying this important clinical phenomenon.

## Materials and Methods

### Ethical statement

Ethical and administrative clearances for the use of baboons in this study were obtained from the Ministry of Scientific Research and Innovation of Cameroon (Research permit #028/MINRESI/B00/C00/C10/C12). The animal procedures were conducted in accordance with the guidelines with animal care and use committee at the National Institutes of Health (USA) and University of Georgia, Athens, USA. Ethical clearance for the involvement of human subjects in the production of infective larvae was obtained from the Institutional Review Board of the Medical Research Station of Kumba, Cameroon. All volunteers were handled according to the Helsinki declaration on the use of humans in biomedical research. The use of non-human primates for research was approved by the Committee on the Ethical Use of Animals in Research (CEUAR) within the Research Foundation for Tropical Diseases and Environment (REFOTDE), Cameroon. All relevant aspects of the International Primatological Society (IPS) 2007 guidelines on the acquisition, care and breeding of non-human primates for research were followed.

### Animals

Baboons of both sexes were trapped in different parts of Cameroon according to IPS standard accepted procedures These animals were transported to the animal facilities in the Tropical Medicine Research Station, Kumba, South West Region and quarantined for a period of two months during which they were pre-screened for a panel of natural infections (loiasis, other blood-borne parasites, and intestinal worms). Each animal was observed daily by the veterinary staff to ensure that they were healthy, and any animal found to be ill was immediately given appropriate treatment, both in the quarantine period and during the main study period.

The animals were housed individually in large custom built cages that allowed the animals to move about freely and be allowed to display their normal repertoire of locomotor behavior (walking, climbing, running, jumping and swinging) by providing them with vertical climbing surfaces and perches. Horizontal surfaces were also provided to allow them to rest comfortably and perform their social interactions such as sprawling during grooming. The housing facility was well aerated and equipped with a system that provided water ad libitum for each animal. Each baboon’s behavior was regularly monitored to identify any indications of poor welfare. Baboons received a diet of food that mimicked their natural diet (leaves, grass, roots, bark, flowers, fruit, lichens, tubers, seeds, mushrooms, corms, and rhizomes). They were also fed a supplement of a nutritionally complete commercial-available diet.

The health and well-being of the baboons were regularly assessed during the study by an animal welfare officer who advised on matters such as disease prophylaxis, zoonoses, anesthesia, and methods of humane euthanasia and provision of health certificates. All measures were taken to minimize suffering during capture, captivity and experimentation. Health screening of workers in contact with the baboons was performed regularly to prevent animal losses from diseases transmitted from humans to baboons as well as zoonotic transmission of disease from baboons to workers. A total of 15 animals (6 males, 9 females) were used in this study with each animal being given a project animal number (BAB-1 to BAB -15).

### Splenectomy

Splenectomy was carried out by a licensed veterinarian following previously published procedures [17,18]. The animals were anaesthetized using an intra-peritoneal injection of 4 mg of betamethasone (Septon, Europe) and 5 mg ketamine (Imalgene, Merial, France); 2mg/kg morphine sulphate (Hamelin Pharmaceuticals Ltd, UK) as also added to the administration as an analgesic. Spleens were removed in approximately 25 minutes under aseptic conditions, the skin wound sutured and disinfected with an antibiotic spray (2.0g Chlortetracycline, 0.5g Gentian violet, 100 mL excipient), and the incision site bandaged. Dressing were changed daily and the animals given a daily 1 mL injection containing 1.2 million units of penicillin and 5 mg of streptomycin with 1 mL of anticoagulation factor (Vitamin K) for a week post-surgery. The surgical wound was dressed daily using an antimicrobial and insect repellent (Veto Spray—Vétoquinol) to protect against flies. The sutures were removed 7 to 8 days after splenectomy. The general welfare of the animals was monitored is on a daily basis for a period of approximately 2 months before infecting the animals with *L*. *loa*.

#### Production of *Loa loa* infective larvae

Female blood-sucking *Chrysops* were caught whilst feeding naturally on *L*. *loa* microfilariaemic human donors. The current standard treatment for loiasis (albendazole, 200mg twice daily for 3 weeks) was administered to all participating donors immediately after the fly feeding procedure. Each fly was kept individually in a 50mL falcon tube prepared to provide suitable conditions for their survival. Each tube was packed with tissue paper to about three quarter its length, a hole made in the cork in which a cotton ball soaked in sugar was placed to feed the fly; a piece of gauze was placed between the cork and tube. The tubes were transported to the laboratory in a cold box. The flies were then maintained at 25–28°C and a relative humidity 77–88% in the insectarium of for 10 to 12 days to allow the maturation of microfilariae into infective larvae within the flies; they were fed with 15% sucrose daily. After this incubation period the flies were dissected in Roswell Park Memorial Institute (RPMI-1640) dissection medium, and the *L*. *loa* parasites present isolated and concentrated in depression wells into batches of 600 L3s; 10–20 L3s were isolated from each fly.

#### Infection and sample collection

For infection and blood sample collection each animal was anesthetized using 0.1mL ketamine. Parasites (600 L3s suspended in 0.3mL RPMI-1640 medium) were inoculated subcutaneously, using a 1mL syringe with a 21-gauge needle, into the anterior portion (inguinal region) of the hind leg. A 10 mL venous blood sample was collected under anaesthesia and aseptic conditions from the femoral vein of each animal for the parasitological, haematological and biochemical analyses. This was carried out at the time of infection and monthly for the first 6 months post-infection and then at two month-intervals up to 22 months.

#### Parasitological analysis

Calibrated thick blood smears for parasitology were prepared by spreading a 50 μL venous blood sample from a 75 μL non-heparinised capillary tube, onto a clean slide over an area of 1.5 cm x 2.5 cm. The identity of each animal was marked on each slide and the blood films were allowed to dry free from dust and flies. Slides were then de-haemoglobinised in tap water for 5 to 10 minutes, fixed with methanol for 1 minute and stained with 10% Giemsa for 45 minutes. The slides were examined under bright field microscopy x10 magnification and microfilariae of *L*. *loa* were identified [22] and quantified, counts being expressed as microfilariae per millilitre (mf/mL) of blood. The blood films were also examined for *Plasmodium* species. Fecal samples were collected and examined at the same time as the blood tests were carried out, with the presence of cysts, oocysts and eggs of intestinal helminths all recorded [23].

#### Haematological and biochemical analysis

All blood samples were processed by centrifugation at 2500–3000 rpm for 10 minutes to obtain serum; the serum samples were divided for either biochemical analysis or haematological parameters. The biochemical parameters were obtained using spectrophotometric kits obtained from HUMAN (www.human.de, Germany) as per the manufacturer’s instructions. The parameters quantified included liver function enzymes (serum glutamate-pyruvate transaminase—SGPT, serum glutamate-oxaloacetate transaminase-SGOT, and serum γ-glutamyl transferase—γ-GT), a kidney function test (creatinine); electrolytes- (calcium, potassium, and glucose). The hematological parameters measured were haemoglobin (Hb), red blood cell count (RBC), total white blood cell count (WBC), and white cell differential count. The differential count was carried out identifying values for neutrophils, eosinophils and mononuclear cells (i.e. lymphocytes and monocytes). These were all determined as described by Cheesbrough [23], except for the haemoglobin levels which were measured spectrophotometrically using Drabkins’s solution (potassium ferricyanide, BDH Chemicals, VWR International).

Data on those haematological and biochemical parameters whose values that did not vary significantly different from normal are described in the S1 File.

### Data analysis

The normal ranges for various blood parameters in baboons used for comparison were those provided by the Association of Primate Veterinarians Primate Formulary (1999) as listed for baboons housed individually in large custom cages. The data were entered into Epi Info version 3.5.3 (C.D.C. Atlanta, GA, USA) and analysed using the Software Package SPSS version 20. Descriptive statistical analyses were performed to compute the mean, median and standard deviations of *Loa* microfilarial counts, different haematological and biochemical parameters in the general study group, and in both males and females. Graph PadPrism software was used to draw the scatter plots comparing microfilariaemia and the different haematological and biochemical parameters to test for any association. The Kruskal and Wallis test was used to test for significant differences in levels of microfilariaemia, haematological and biochemical parameters before inoculation and at different time points of observation in the general study population. The Mann-Whitney test was used to test for significant differences in the different haematological and biochemical parameters between males and females. The Jonchkeere-Terpstra (J-T) test was used to test for any trend of linearity in the different parameters at different months. All tests were performed to a 5% significance level.

## Results

### General health of the animals

All fifteen animals appeared well fed and remained healthy throughout the study. The surgical incision sites post-splenectomy all healed without any evidence of infection, and no animal showed fever nor any intestinal disturbances during the study. The pre-study screening did not detect the presence of malaria or any intestinal parasites, in the test animals.

### Time-course of *Loa* microfilariaemia

**Fig 1:**
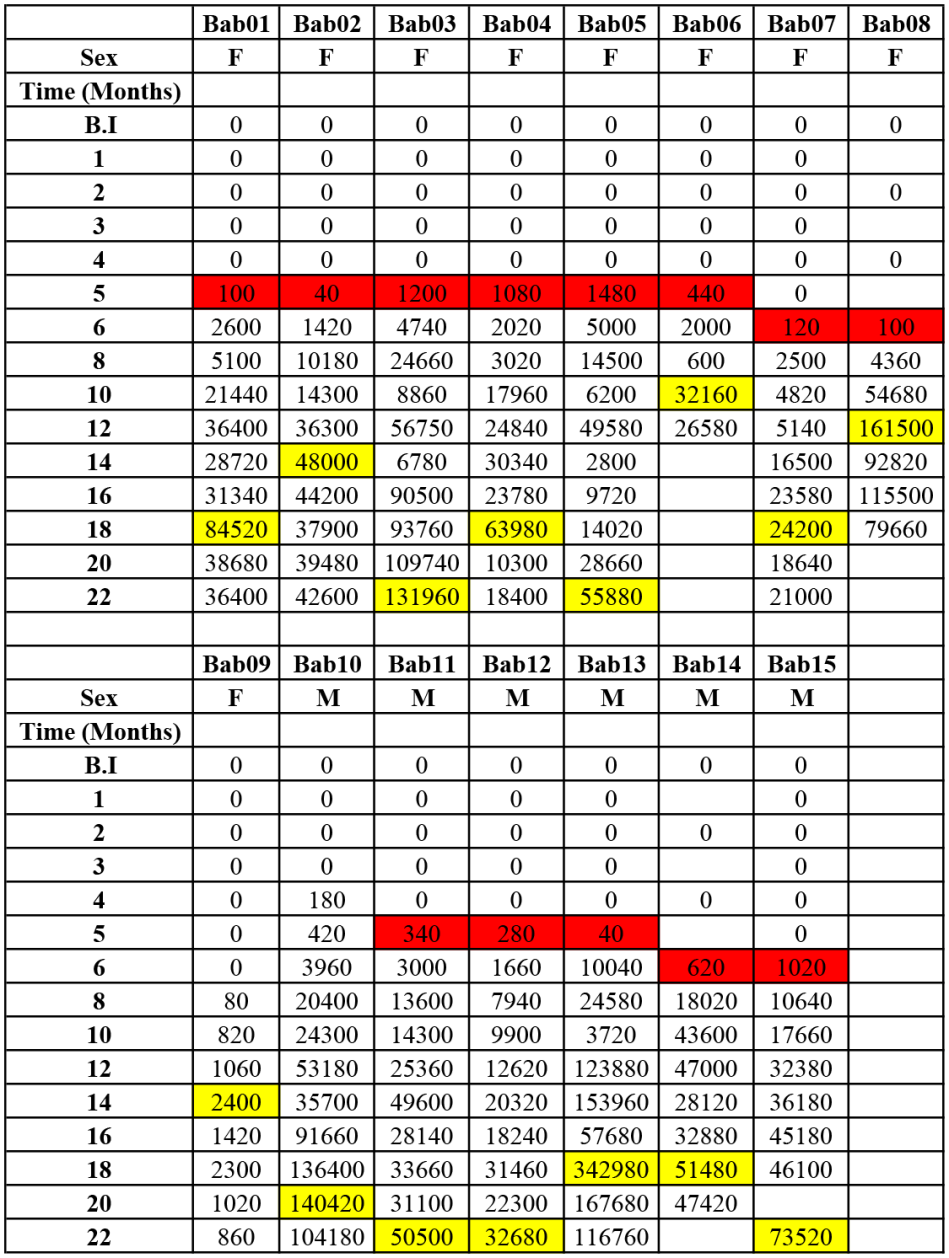
Variation in microfilariaemia (mf/ml) with respect to sexes. Cells highlighted in red show the month microfilariae starts being observed in the blood of each animal (patency). Cells highlighted in yellow show the month the maximum microfilaria load was observed in each animal. Empty spaces indicate dates were data was not obtained for varied reasons e.g. sickness from non-parasitological conditions, etc.

**Fig 2:**
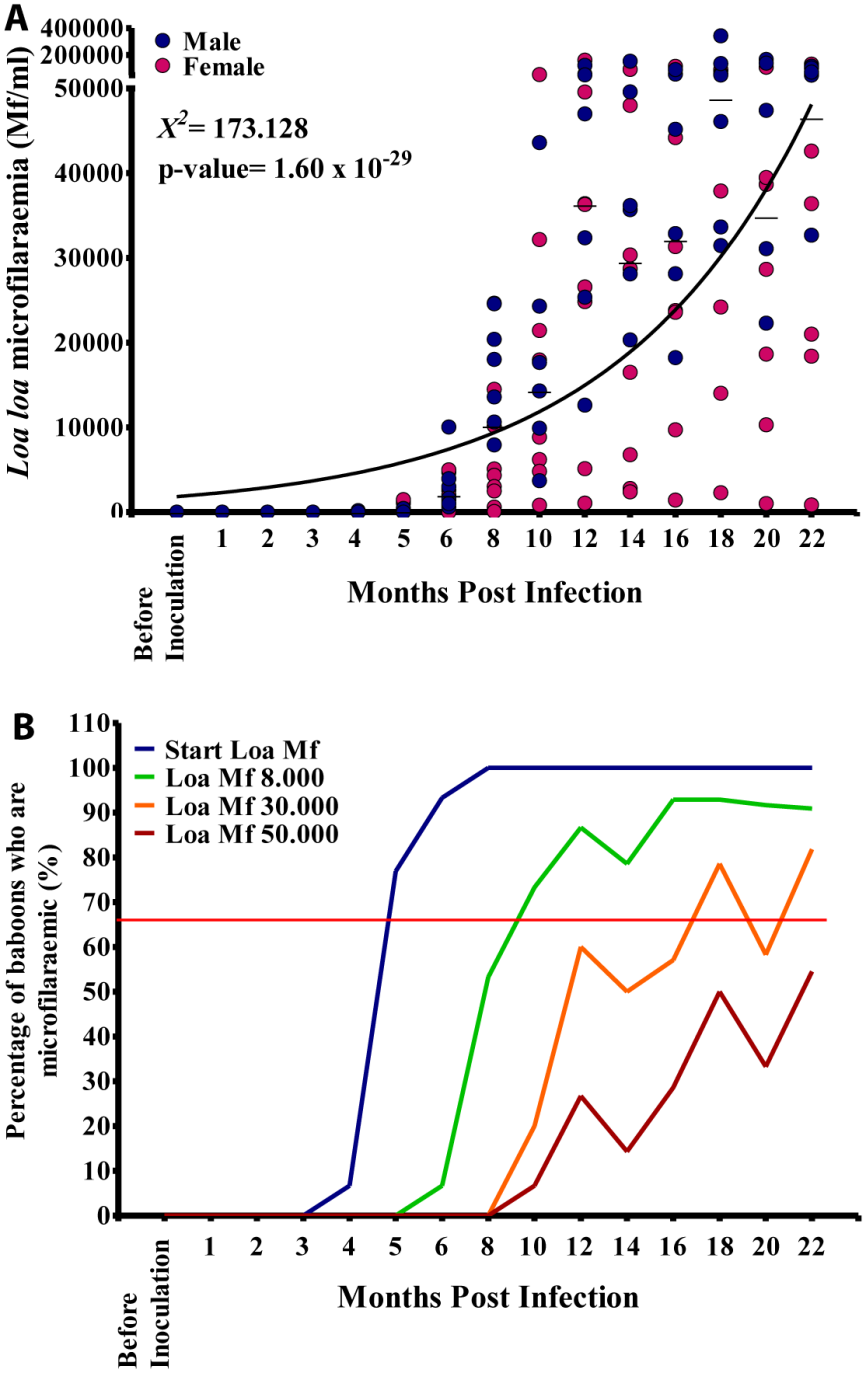
Microfilariaemia in splenectomised baboons. A. Time course of microfilariaemia in 15 baboons experimentally infected with human *Loa loa*. B. Proportions of baboons who are *Loa loa* microfilariaemic at various times during infection.

The pre-patent period of this infection in baboons ranged from 4–8 months, with a median of 5 months. 1 of 15 (6.7%), 9 of 15 (60%) baboons, 4 of 15 (26.7%), 1 of 15 (6.7%) had pre-patent periods respectively of 4, 5, 6, and 8 MPI (Figs 1 and 2). The pre-patent period for males ranged from 4–6 months (median 5 months) with 3 out of 6 males (50%) becoming patent at 5 months post infection (Fig 1). The pre-patent period of females ranged from 5–8 months (median 5 months) with 6 out of 9 females (66.67%) becoming patent at month 5 post infection (Fig 1). There was no significant difference between the median pre-patent period of males and that of females (p = 0.504). The month at which each baboon started having microfilariae in blood (pre-patency period) and the month at which each baboon developed its highest microfilariaemia is shown in Fig 1.

### Kinetics of *Loa* microfilariaemia

Animals were followed up for 22 months by which time all animals had become patent (Fig 2). By month 4 post inoculation (MPI) about 7% of infected baboons had microfilariae present in their circulation, and by month 5 MPI >70% of infected animals had developed microfilariaemia. By month 8 MPI all animals were parasitologically positive (Fig 2B). Generally, the mf increased steadily in all animals from the onset of patency to reach a median of 48,790 mf/mL by month 18. The mf loads at different time points during the course of infection was highly variable (Fig 2A). Male baboons generally developed higher microfilariaemia than females (Fig 1), although this difference was not statistically significant (p = 0.06). By 6 MPI about 7% of animals had developed mf loads >8,000 mf/mL; at 8 MPI 50% of them had developed mf loads >8,000 mf/mL, and at 10 MPI >70% of animals had developed mf loads >8,000 mf/mL (Figs 1 and 2A).

With regards especially high blood microfilarial loads, about 20% of animals had developed mf loads >30,000 mf/mL by 10 MPI. At 14 MPI, 50% of infected animals had developed mf loads that were >30,000 mf/mL and at 18 MPI >70% of infected animals had developed mf loads >30,000 mf/mL (Fig 2B). By 10 MPI about 7% of infected animals had developed extremely high blood microfilarial loads of >50,000 mf/mL and by 18 MPI almost 50% of them had developed these very high microfilarial loads (Fig 2B).

### Changes in haematological parameters during the course of infection

**Fig 3:**
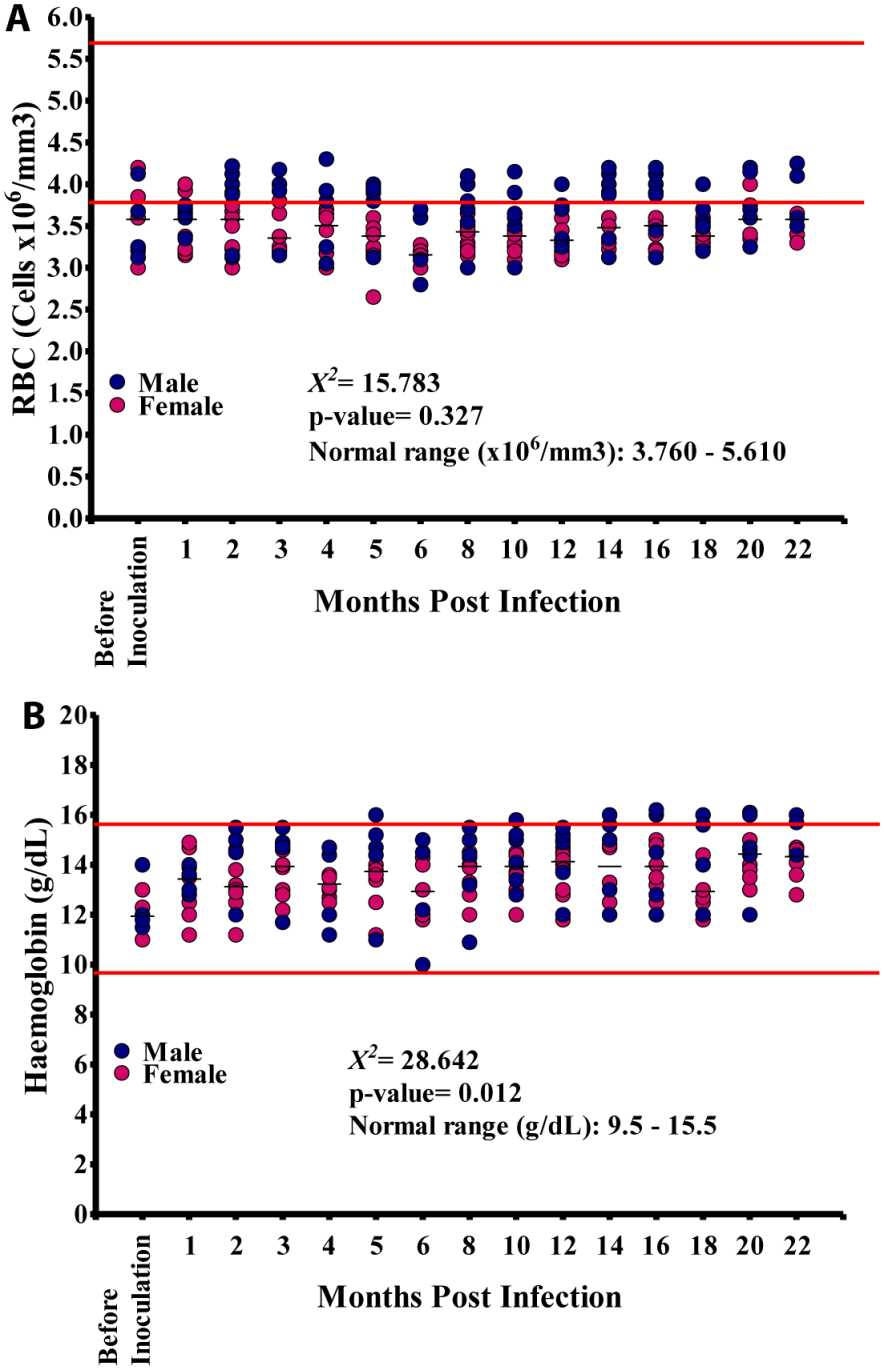
Red blood cell (RBC) and hemoglobin values in the 15 baboons during the course of the infection. A. Variation in the RBC count during the course of infection. B. Variation in haemoglobin levels during the course of infection.

RBC counts ranged from 2.65–4.3 x10^6^ cells/mm^3^ (median 3.48 x10^6^ cells/mm^3^; NV = 3.76–5.61 x10^6^ cells/mm^3^). Again males differed significantly from females in RBC values (p<0.001). Most RBC values recorded in all animals were below the NV at the different time points (Fig 3A). Hemoglobin (Hb) values ranged from 10–16.2g/dl (median: 13.90g/dl) essentially close to normal values (NV) of 9.5–14.5g/dl (Fig 3B), with a slight upward trend during the infection (see S1 File). There was significant difference between males and females: Hb values in males ranged from 10–16.2g/dl (median: 14.4g/dl) whilst for females the values were 11–15.2g/dl (median 13.6g/dl); these were significantly different (p<0.01).

**Fig 4:**
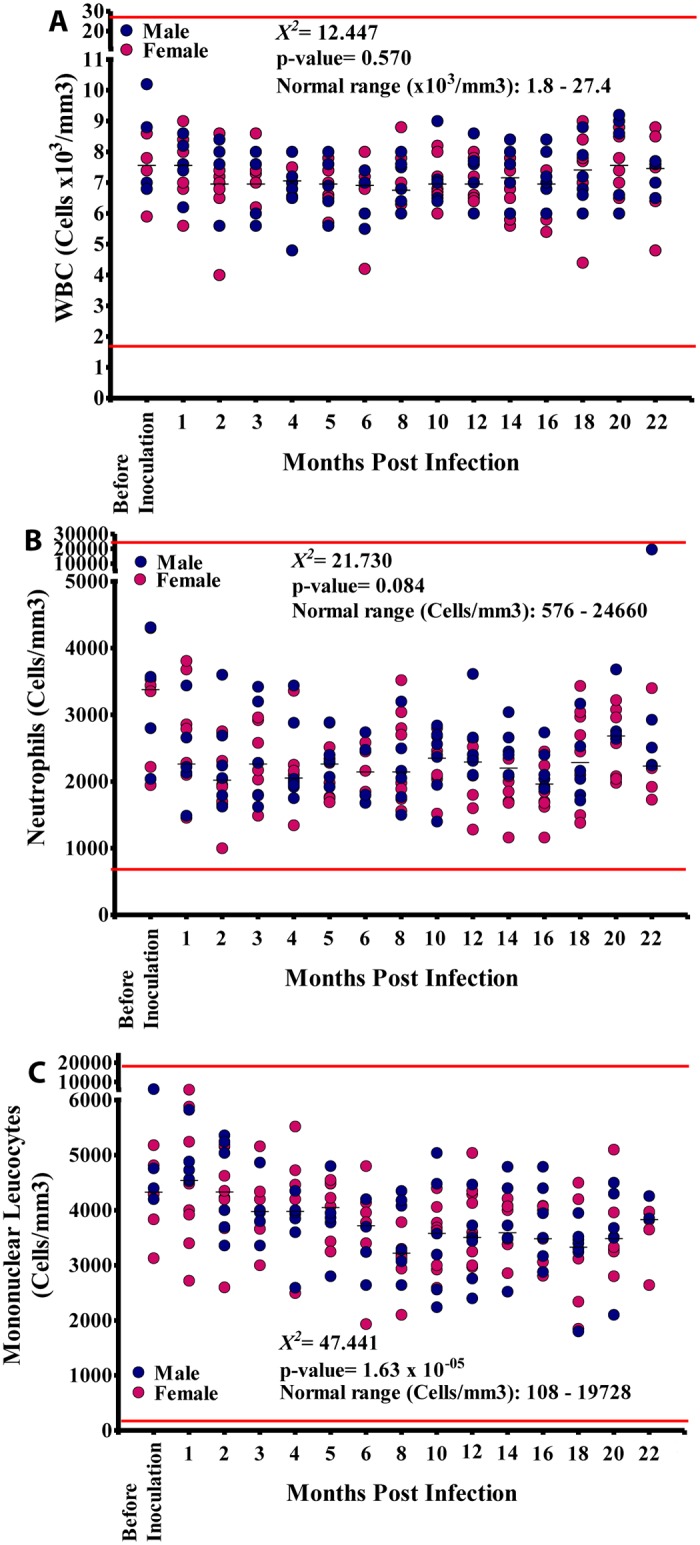
White blood cell (WBC) counts during infection. A. Total WBC. B. Absolute neutrophil counts. C. Absolute mononuclear (monocyte and lymphocyte) counts.

The total white cell counts were generally all within the NV (Fig 4A). Absolute neutrophil counts also stayed within NV ranging from 1,000–19,500 cells/mm^3^ (Median: 2,250 cells/mm^3^) (Fig 4B). Absolute mononuclear (lymphocyte + monocyte) cell counts ranged from 2,400–34,760 cells/mm^3^ and were within the NV (see S1 File) although was a marked variation between different time points (Fig 4C). with a median of 3,796 cells/mm^3^ (NV: 810–19,728 cells/mm^3^). The values for males were 1,800–39,000 cells/mm^3^ (median: 3,788 cells/mm^3^) while in females these counts ranged from 1,848–6,030 cells/mm^3^ (median: 3,810 cells/mm^3^). These mononuclear cell counts did not vary significantly between males and females (p = 0.904). Absolute counts at different time points however did vary significantly (p<0.001) and showed a significant linear trend (p<0.001). Mononuclear counts recorded were within the NV (Fig 4C).

**Fig 5:**
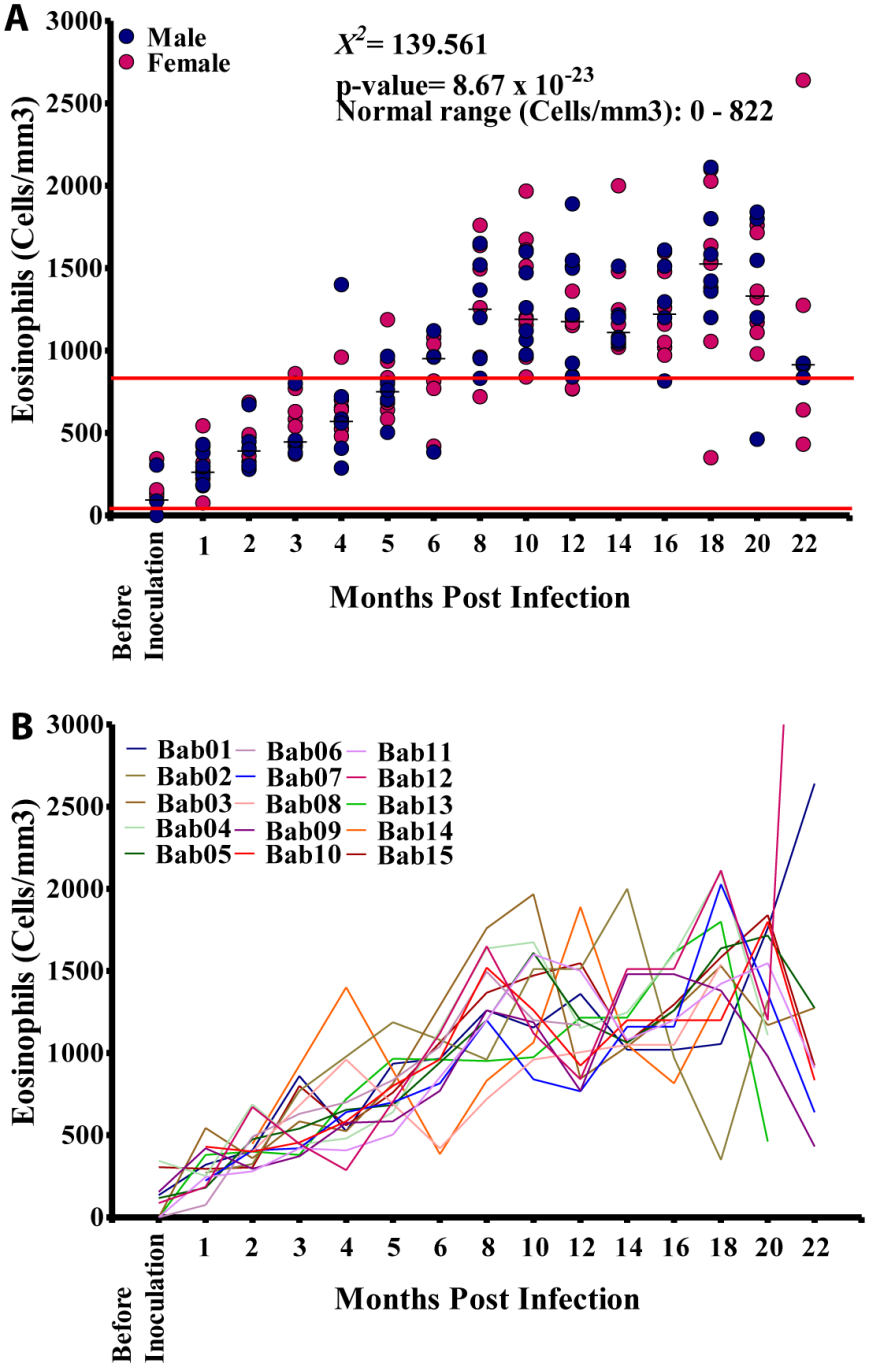
Eosinophil profiles during infection. A. Absolute eosinophil counts in the 15 splenectomised infected baboons. B. Absolute eosinophils counts in the individual animals.

Absolute eosinophil counts ranged from 0–6,500 cells/mm^3^ (Median: 978 cells/mm^3^; NV: 0–822 cells/mm^3^). Absolute eosinophil values in males ranged from 0–6,500 cells/mm^3^ (Median: 1,013 cells/mm^3^) while in females absolute eosinophil levels values ranged from 0–2,640 cells/mm^3^ with a median of 972 cells/mm^3^; these were not significantly different (p = 0.473). Eosinophil counts at different time points, however did vary significantly (p<0.001) and showed a general increase over the 18 months studied. All animals recorded absolute eosinophil values out of the normal range at different time points (Fig 5A) The absolute eosinophil results for each animal are given in Fig 5B. Basophils were not identified in these study samples.

### Changes in biochemical parameters during the course of infection

**Fig 6:**
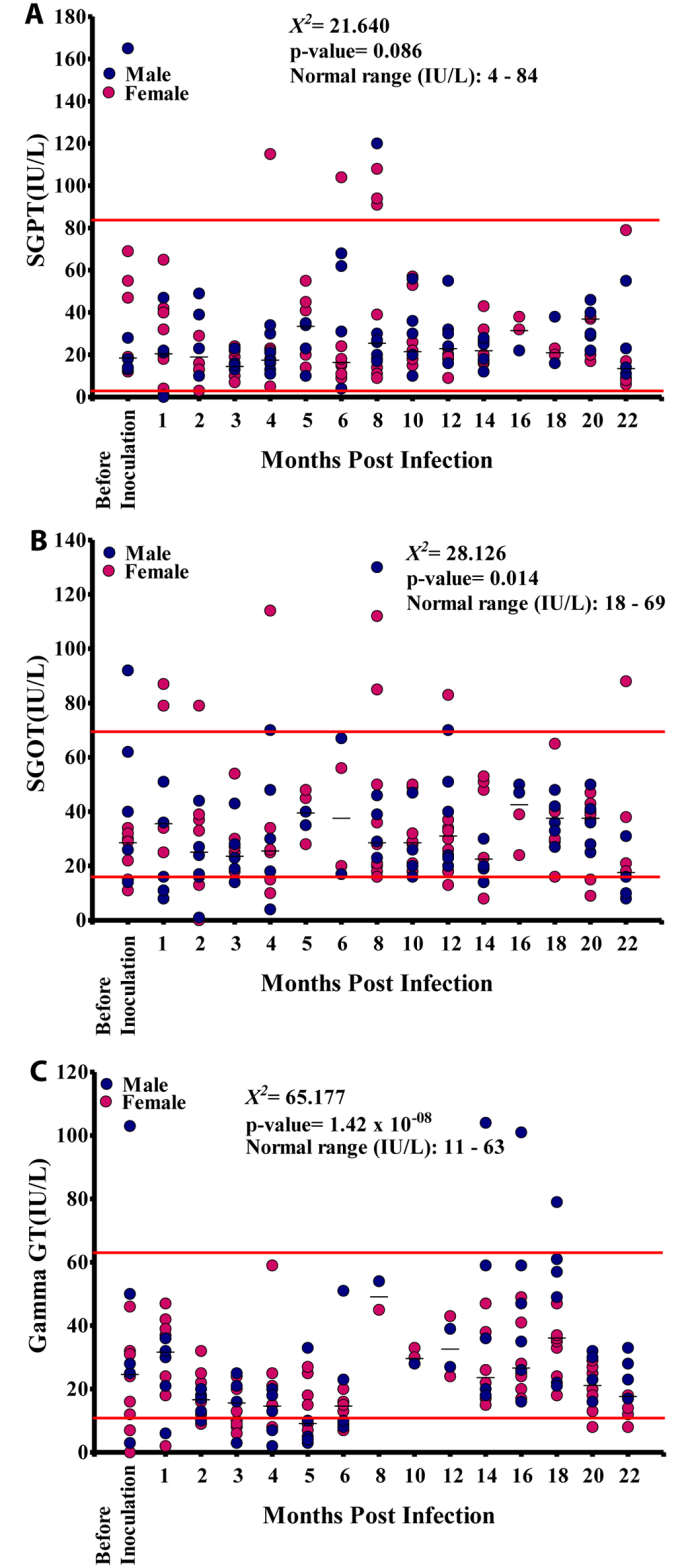
Enzyme levels in the 15 baboons infected with L*oa loa*. A. Serum glutamic pyruvic transaminase (SGPT) levels. B. Serum glutamic-oxaloacetic transaminase (SGOT) levels. C. gamma-glutamyl transferase (GGT) levels.

There was no significant difference in SGPT values between males and females (p = 0.342), nor did the SGPT values at different time points vary significantly (p = 0.086) or show a significant linear trend (p = 0.110) with the majority of SGPT values being within the NV (Fig 6A). The SGOT values at different time points varied significantly (p<0.05) although there was no significant linear trend (p = 0.356). The majority of SGOT values were within the NV, even though all baboons except BAB 04 showed SGOT values both below or above the NV at different time points (Fig 6B). The γ-GT values at different time points varied significantly (p<0.001), and there was a significant linear relationship between microfilariaemia and the duration of infection (p<0.05). However, most of the γ-GT values were within the NV (Fig 6C).

**Fig 7:**
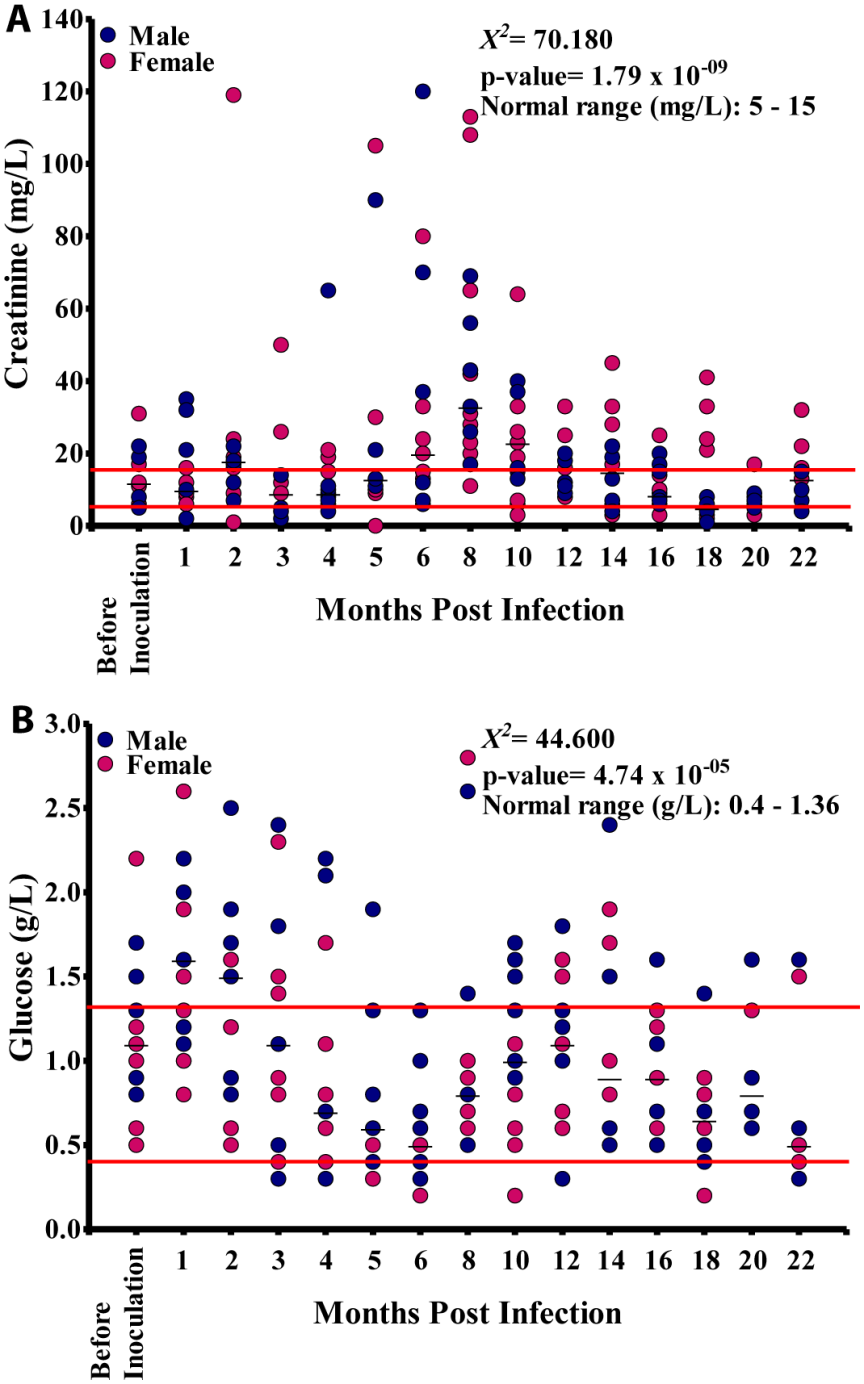
Blood compound biochemistry during *Loa loa* infection. A. Creatinine levels in 15 baboons. B. Glucose levels in these animals.

The creatinine values at different time points varied significantly (p<0.001), although the values did not show any significant linear relationship with the duration of infection (p = 0.068). Most of the creatinine values were above the NV at the different time points (Fig 7A). Glucose values varied significantly (p<0.001) at different time points, and there was significant linear relationship between blood glucose level and the duration of infection (p<0.001). Majority of the glucose values were within the normal range, although all baboons showed glucose values out of the normal range at different time points (Fig 7B).

**Fig 8:**
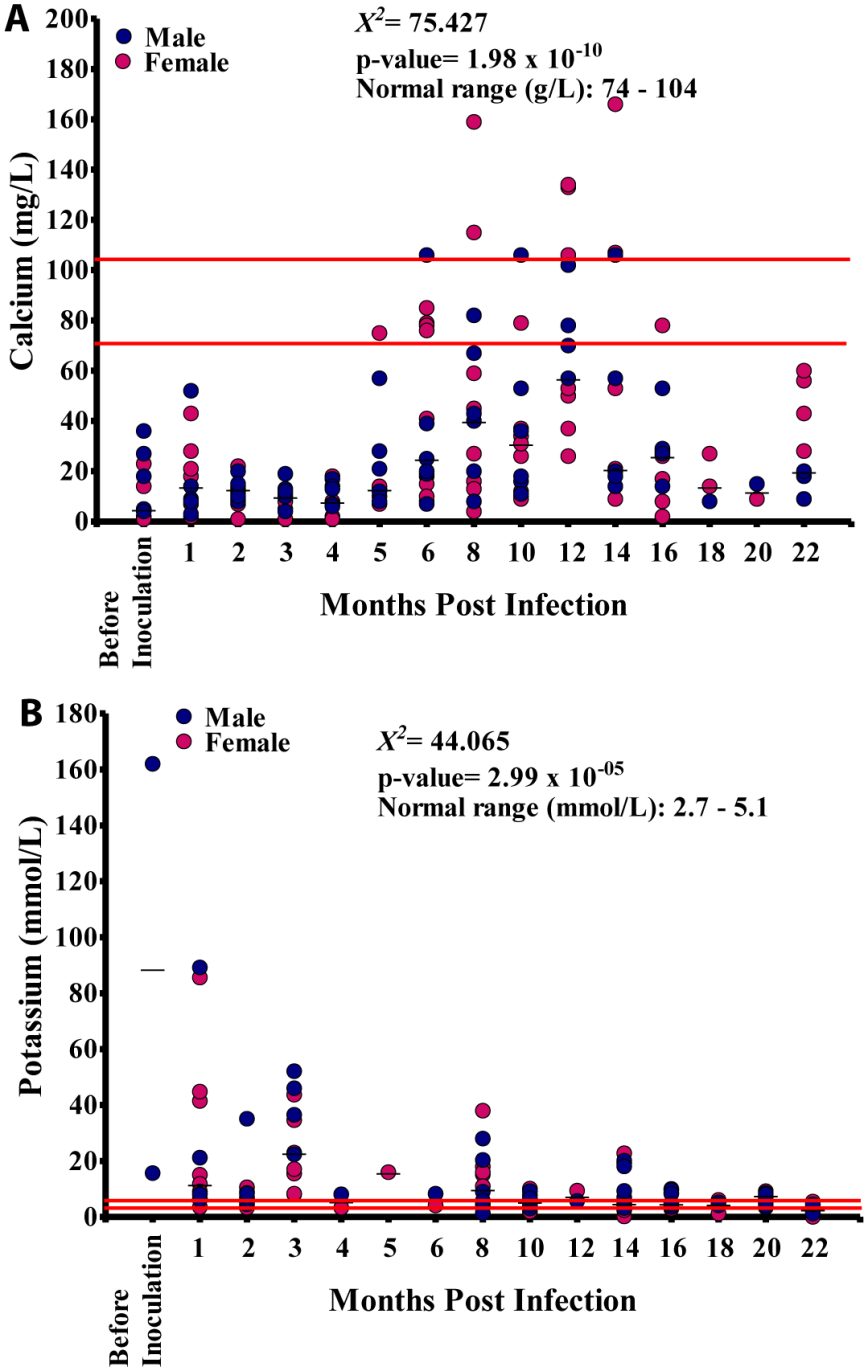
Blood elemental biochemistry during *Loa loa* infection. A. Calcium levels in 15 baboons. B. Potassium levels in these animals.

Calcium values varied significantly at different time points (p<0.001) and showed a significant linear relationship with the duration of infection (p<0.001). Majority of the calcium values in all animals at different time points were below the NV (Fig 8A). The potassium values at different time points varied significantly (p<0.001), and showed a significant negative linear relationship with the duration of infection (p<0.001). The majority of the potassium values in all animals were out of the NV (Fig 8B).

### The relationship between microfilariaemia and the haematological/biochemical parameters

**Fig 9:**
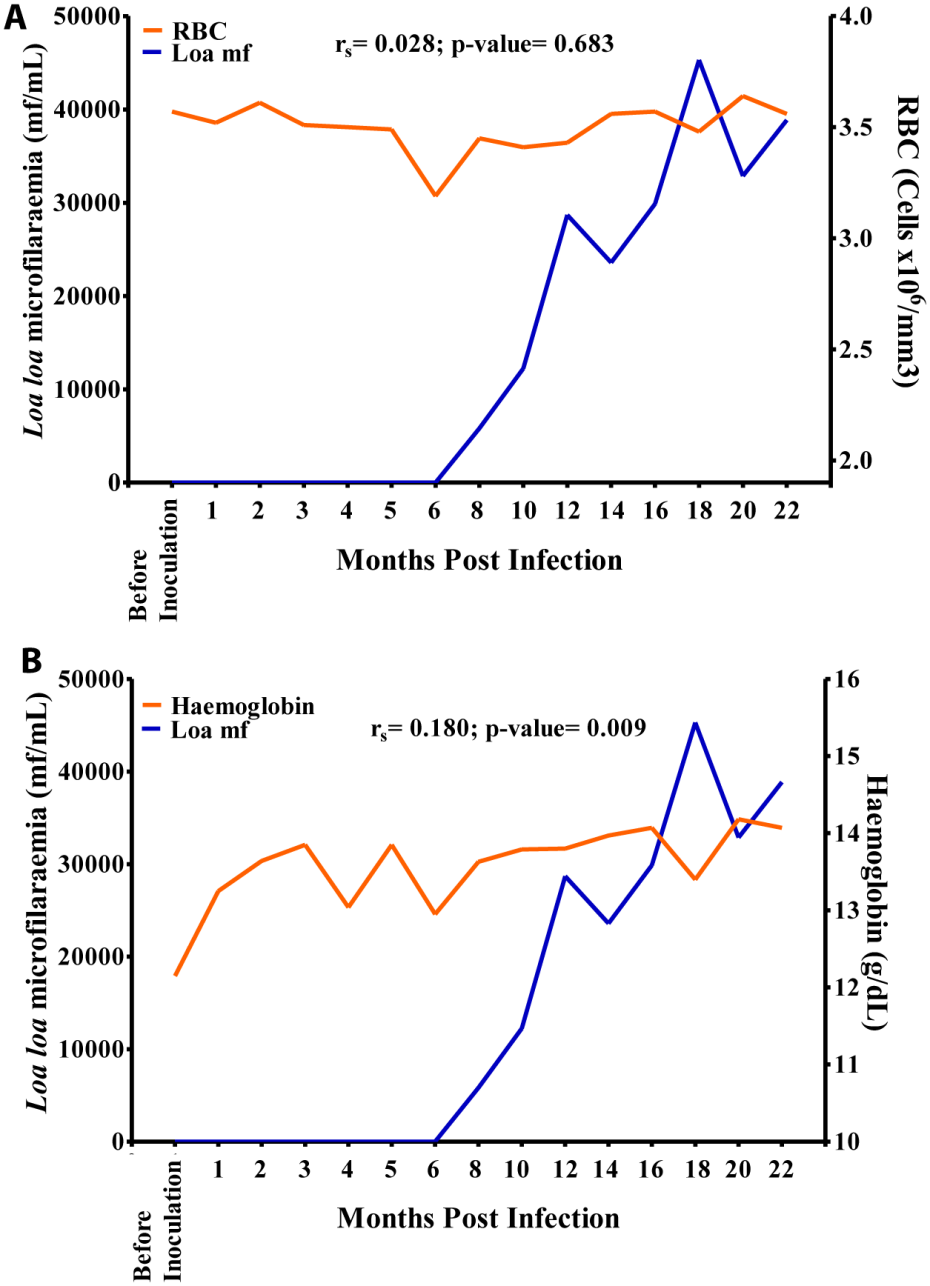
Relationship between microfilariaemia red blood cell (RBC) and haemoglobin values during the period of infection. A. Relationship between microfilariaemia and RBC. B. Relationship between microfilariaemia and haemoglobin.

**Fig 10:**
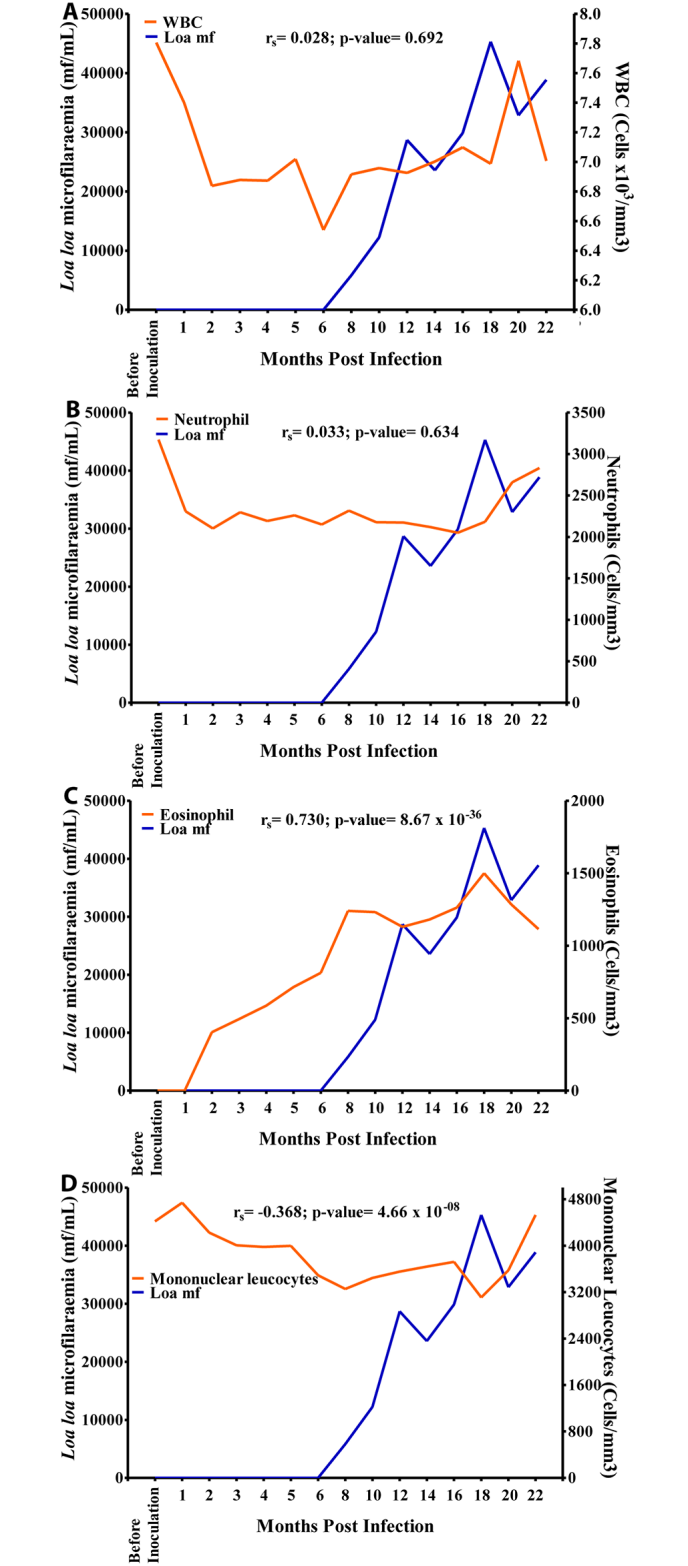
Relationship between microfilariaemia and white blood cell (WBC) counts during the period of infection. A. Relationship between microfilariaemia and total WBC. B. Relationship between microfilariaemia and neutrophil. C. Relationship between microfilariaemia and eosinophil. D. Relationship between microfilariaemia and mononuclear cells.

Hb values showed a slow increase from 12.4 g/dL before inoculation to 14.1 g/dL at 3 MPI after which the values dropped to 13.1 g/dL at 6 MPI and increased steadily over time to 14.1 g/dL at 16 MPI where a slight dropped to 13.3 g/dL was noticed as mf culminated at 18 MPI. Overall, there was a positive significant association (r = 0.180, p>0.05) between mf and Hb values (Fig 9B). The eosinophil count increased sharply from 0 cells/mm^3^ before inoculation to 1,500 cells/mm^3^ at 18 MPI when the mf reached its highest level. Overall, there was a strong positive significant association (r = 0.730, p<0.001) between eosinophil and mf (Fig 10C). The mononuclear count decreased steadily from 4,800 cells/mm^3^ at 1 MPI to 3,800 cells/mm^3^ at 16 MPI from where its values dropped slightly to 3,200 cells/mm^3^ at 18 MPI when the mf peaked. Overall, there was a negative significant association (r = -0.368, p<0.001) between mf and mononuclear counts (Fig 10D). There was no significant association between mf and RBC, WBC and neutrophil (Figs 9A, 10A and 10B).

**Fig 11:**
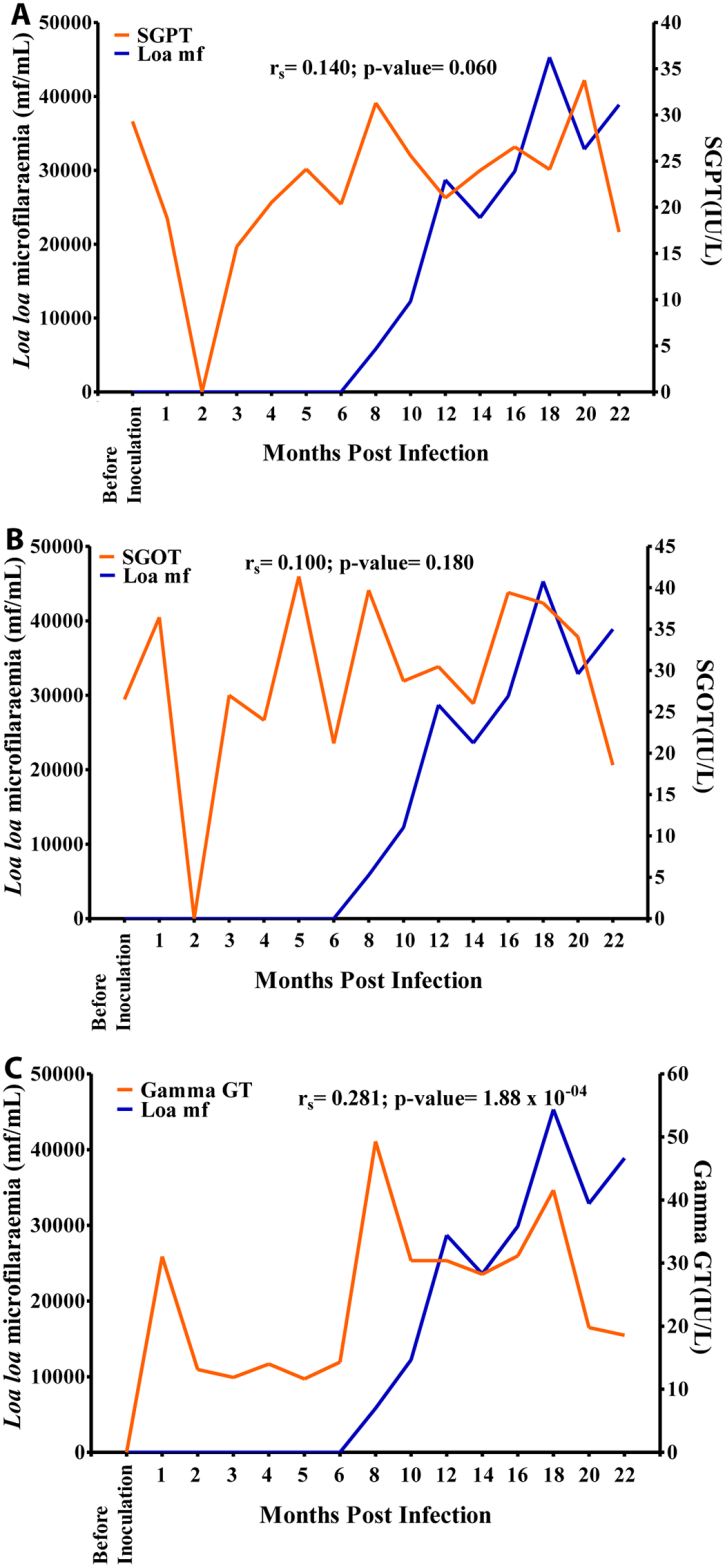
Relationship between microfilariaemia and blood elemental biochemistry during the period of infection. A. Relationship between microfilariaemia and calcium. B. Relationship between microfilariaemia and potassium levels.

The enzymes SGPT and SGOT did not show any significant association with mf (Fig 11A and 11B). Overall, there was a positive significant association (r = 0.281, p<0.001) between γ-GT and mf (Fig 11C). γ-GT values increased sharply from 0 IU/L before inoculation to 50 IU/L at 8 MPI, then decreased slightly over time to 29 at 14 MPI after which time its values decreased gradually over time to 42 IU/L at 18 MPI when mf peaked and then dropped again.

**Fig 12:**
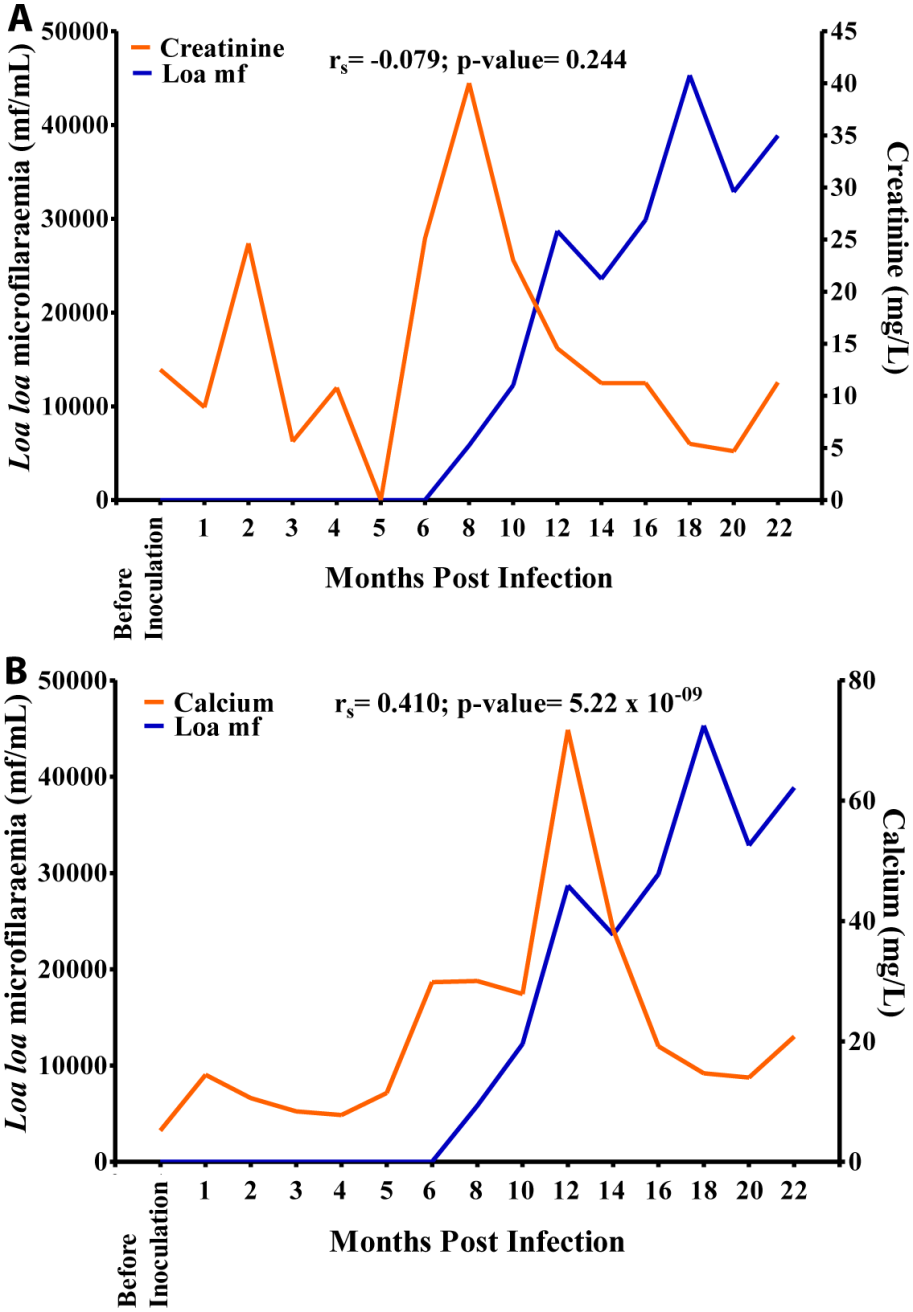
Relationship between microfilariaemia and blood compound biochemistry during the period of infection. A. Relationship between microfilariaemia and creatinine. B. relationship between microfilariaemia and glucose.

There was a negative, non-significant, association between mf and creatinine (Fig 12A). Glucose values decreased gradually from 1.5 g/L at 1 MPI to 0.6 g/L at 6 MPI from where the values increased slightly to 1.1 g/L at 10 MPI after which its values decreased to 0.6 g/L at 18 MPI. Overall, there was a negative significant association (r = -0.171, p<0.05) between glucose and mf, (Fig 12B).

**Fig 13:**
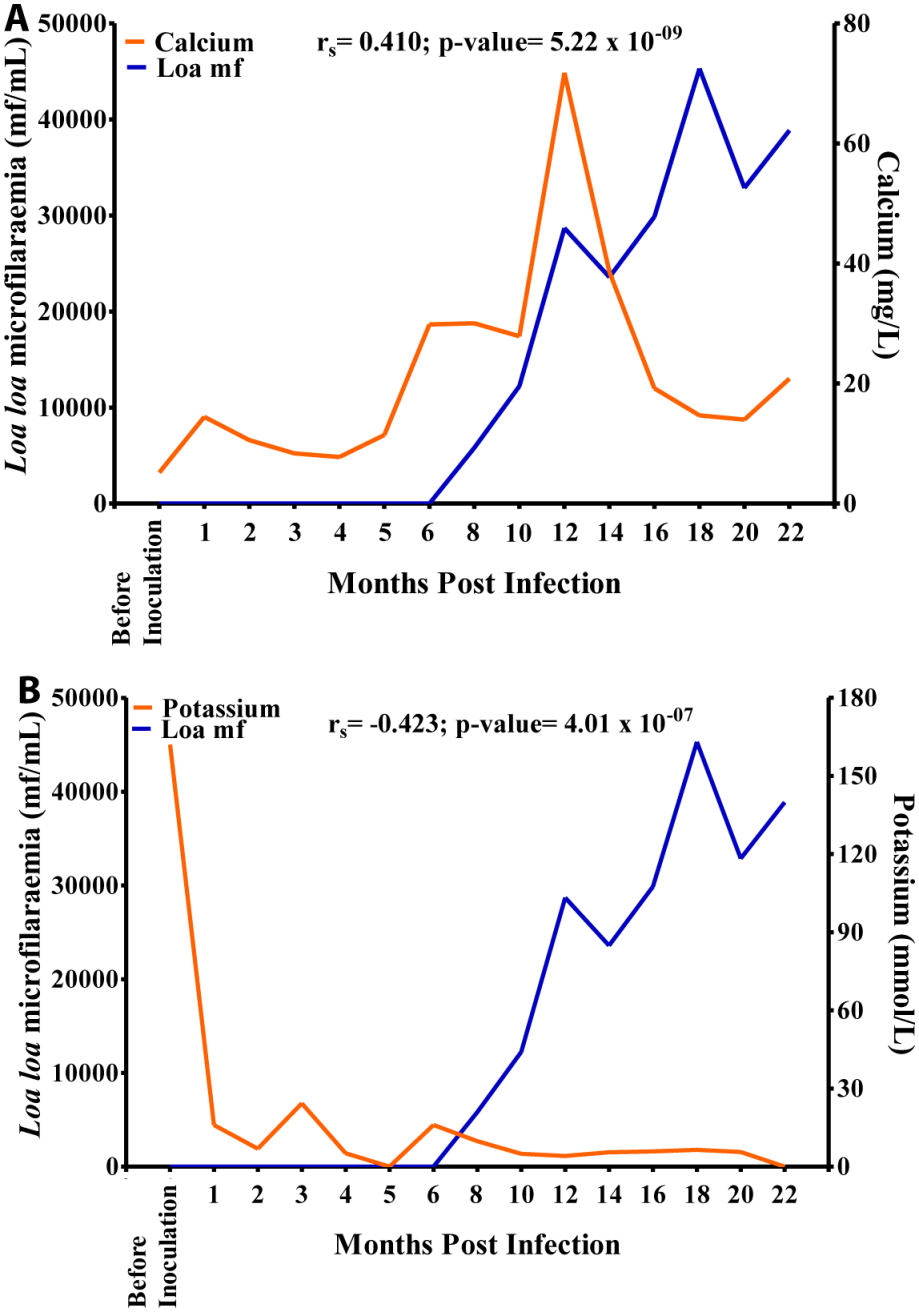
Relationship between microfilariaemia and enzymes during the period of infection. A. Relationship between microfilariaemia and SGPT. B. Relationship between microfilariaemia and SGOT. C. Relationship between microfilariaemia and gamma-GT.

Calcium values increased steadily over time from 9 mg/L before inoculation to 25 mg/L at 10 MPI at which point it increased sharply to 74 mg/L at 12 MPI after which its values decreased steeply over time to 15 mg/L at 20 MPI. Overall, there was a positive significant association (r = 0.410, p<0.001) between calcium and mf (Fig 13A). Potassium values plummeted from 170 mmol/L before inoculation to 5 mmol/L at 18 MPI when the mf peaked. Overall, there was a negative significant association (r = -0.423, p<0.001), between mf and potassium (Fig 13B).

## Discussion

This study extends the findings made a number of years ago showing that primates can be infected with human derived *L*. *loa*. In this present study the time course and kinetics of *Loa* microfilariaemia in the baboon (*Papio anubis*) were characterized in detail showing that all infected spelectomized animals become patent, and with the majority doing so by 5 months after inoculation. This finding is comparable to the earlier observations of Duke and Wijers [11], Duke [17], Orihel and Moore [12, 24] and Dennis et al [25], with a major difference being that in this present study the animals were splenectomised before inoculation. However, the pre-patency periods for the infection in the animals splenectomised before inoculation with *Loa* and those splenectomised after infection are the same suggesting that the spleen plays only a minor role in determining the pre-patency of these animals. The extended pre-patent period (8 months) observed in one baboon cannot be readily explained or attributed to anything other than a peculiarity in the individual host-parasite relationship. All the animals in this present study were able to attain very high microfilarial loads.

Although the spleen appears not to influence to any great degree the development of patency this organ may however have a more significant role in maintaining patency and possibly the microfilarial load eventually reached, i.e. be involved in the rate of removal of microfilariae from the circulation. It is also possible that after splenectomy there may be regeneration of functional splenic tissues; this possibility, and the previously reported data from mandrills, adds to the contention that the spleen is likely to be an important player in controlling the load of circulating microfilariae.

These findings indicate that the baboon is a very good experimental model for studying conditions related to high circulating microfilarial loads such as the *Loa* encephalopathy seen post ivermectin treatment. As the time course of *Loa* microfilariaemia steadily increases to reach a peak at month 18 (Fig 2) in a relatively regular manner it is possible to develop reproducible experimental protocols to test various questions related to specific microfilarial loads. It is thus possible, therefore, to test different possible treatment regimes in scenarios that parallel various human microfilarial loads, and can do this beginning at month 5, when baboons start showing microfilariaemia, up until around month 18, when baboons reached their highest microfilariaemia. It was also noted that although microfilariaemia rose steadily in both sexes of these baboons, male baboons often developed a slightly higher microfilarial loads than females. Generally, differences between gender regarding disease occurrence have been related to physiological causes particularly hormonal and genetic ones [26]. Nevertheless, as there were no major differences in the duration of pre-patency period, nor in the maximum attained microfilariaemia between male and female baboons, it is likely that both sexes can be used for this type of experiment.

In this present study we have used a relatively high number of infective larvae (600 L3s) to induce infection compared to that used previously in mandrills. We have found this provides the most consistent infection rate in baboons. The reason for the need for a higher number of L3s compared to those needed to consistently infect mandrills is not clear at this time and it possible that this parasite is more adapted to surviving in mandrills, where lower numbers of larvae have been used, compared to baboons; other technical reasons might be at play here as well.

In humans it has believed that a microfilariaemia of >8,000mf/mL is a marker characteristic of those who develop serious non-neurological conditions for treatment, although >30,000mf/mL has been proposed as the main danger point in humans [5–8] in terms of a risk, with those >50,000 mf/mL regarded as very high risk individuals. This baboon model, with its ability to produce microfilarial loads consistently, is an important tool for understanding in more detail relationships between microfilarial load and pathology. Most animals (13 out of 15–86.67%) of the infected animals developed microfilariaemia of >30,000mf/mL. These findings differ from Dennis *et al*. [25] who observed only low microfilariaemia (250–1,000mf/mL) in splenectomised rhesus monkeys; this difference may be due to the fact that baboons are natural hosts for *L*. *loa* and rhesus are not.

All infected animals develop eosinophilia well above the normal range, a finding expected in a filarial infection as with many helminths [27]. Hyper-eosinophilia and loiasis have been noted in previous studies in humans [28] but not previously described in experimental models of loiasis [29]. The fact that all infected baboons developed a significant increase in eosinophilia prior to the onset of patency and that the levels continued to increase after patency suggests that adult worm antigens and associated cytokines are most likely to be involved in this induction of the eosinophilia [30]. The strong significant positive association between eosinophil levels and microfilarial loads is to be expected in helminth infections [31] as eosinophils are major effector cells in the immune responses to the presence of this parasite. A major question that now remains is whether this significant blood eosinophilia is intimately involved in the pathogenesis of the SAE as has been suggested [9].

It is not clear as to the biological significance of the variations seen here in total red blood cell counts seen at different time points, nor with the positive significant association between microfilariaemia and haemoglobin levels. Although variations occurred in haemoglobin levels at different time points the majority of measured values were within the normal range. As no animals were found to be clinically anaemic, and as the animals were fed on a rich protein-diets, it is likely that the variations in these parameters were not a major characteristic of this infection; this interpretation is supported by the findings of Johnson *et al*. [32] with vervet monkeys (*Chlorocebus aethiops)* in captivity fed on a high protein diet who also did not develop anaemia. The observation that males had a significantly higher concentration of haemoglobin and red blood cells than females maybe due to the greater muscle mass of males [33], or perhaps the menstrual blood loss in females [34]. The increased neutrophil levels are hard to explain without further tissue level investigation of these animals; there were no obvious bacterial or other infections in these animals that could have explained such a neutrophilia. It is noted that neutrophils, eosinophils, as well as monocytes, have all been shown experimentally to be capable of damaging and destroying microfilarial of various species through complement-dependent, IgG mediated, mechanisms [35]; such phenomenon may be involved in the altered neutrophil levels.

The elevations in blood creatinine in some animals before inoculation and their increase during the course of infection could be related to kidney dysfunction [36]. The retention of creatinine in the body likely indicates that the kidneys were failing to efficiently excrete these catabolic products. Studies on filariasis in dogs have demonstrated an increase in serum creatinine levels in dogs infected with *Dipetalonema reconditum* [37]. However, other physiological factors or substances unrelated to the infection may have contributed to this creatinine response since no significant association was seen between the appearance of microfilariae and the rise in creatinine levels. SGPT, SGOT, γ-GT and glucose levels were relatively stable indicating a proper functioning of the liver in these animals. The values were recorded here are in general agreement with Núńez *et al*. [38] who reported minimal changes in liver function for healthy (uninfected) young and adult *Cebus appella* monkeys of both sexes. It should be noted, however, that although the baboons remained healthy throughout the study, the animals in our study often had histories of varied and different diets which could explain variations in liver enzyme activities [39, 40]. It is not clear as to what could have caused the negative significant association between glucose and microfilariaemia; further studies, including the effect of diet, are need to explain this finding. The positive significant association seen between γ-GT and microfilariaemia corroborates the findings of Molina *et al*. [41], where visual hepatic damage and serum levels of γ-GT were significantly positively related in cattle infected with a helminthic parasite. The positive significant association noticed between microfilariaemia and calcium and the negative significant association between potassium and microfilariaemia could probably be due to kidney dysfunction in these animals [42, 43].

Many of the biochemical parameters in this present study were within the normal range; however, it is noted that significant changes in hemoglobin related parameters have been observed with other infections in the baboon, some that can be directly related to the specific infection. For instance, Mustafa *et al*. [44] demonstrated that haemoglobin levels were found to be generally low in baboons infected with *Plasmodium knowlesi* parasites. Al-Tayib [45] demonstrated that infection of baboons with *Balantidium coli* showed severe anaemia and in increased values of SGOT and SGPT well above their normal ranges. The effect of diet with animals kept in captivity must also be considered; many biochemical parameters are known to vary greatly with the diet and a major shortcoming of this present study is that the various biochemical samples taken from these animals were not standardized on a fasting state. However, the three most significant alterations seen here, i.e. the increased eosinophil, creatinine and gamma-GT level, are three parameters that can be argued as most likely be related to the presence of the filarial infection. An intriguing question that emerges now is whether these specific alterations bear any role in the development of the post-ivermectin encephalopathic SAE.

### Conclusion

This study is the first showing parasitological, haematological and biochemical characterization of hyper-microfilaraemic loiasis in the splenectomized baboon (*P*. *anubis*). Parasite pre-patency was between 4–8 months, with the majority (60%) of animals becoming patent 5 months post inoculation; all animals developed patency; Microfilariaemia rose steadily in all animals and culminated at a peak level by month 18 post infection with males showed higher microfilariaemia than females. By month 10 post inoculation >70% of infected animals developed microfilariaemia >8,000mf/mL; by month 18 post inoculation >70% of infected animals had developed microfilariaemia >30,000mf/mL, and 50% of them developed >50,000mf/mL a level where in humans that predisposed for severe adverse reactions post treatment. Significant positive associations were seen between microfilariaemia and eosinophil, haemoglobin, calcium and gamma-GT, whilst there was a negative significant correlation between microfilariaemia and mononuclear leucocytes, glucose and potassium. This model has the potential of helping to understand the mechanism(s) involved in the development of *Loa*-encephalopathy post-ivermectin treatment in heavily *Loa* microfilariaemic humans, and could help in designing improved management of such cases.

## Supporting Information

S1 File Data and descriptions of those haematological and biochemical parameters whose values that did not vary significantly different from normal. (DOCX)
