# Supplementary material for: Parasitological, Hematological and Biochemical Characteristics of a Model of Hyper-microfilariaemic Loiasis (Loa loa) in the Baboon (Papio anubis)
Source: PLoS Negl Trop Dis. 2015 Nov 10;9(11):e0004202. doi: 10.1371/journal.pntd.0004202 (PMC4640546; doi:10.1371/journal.pntd.0004202)
Supplement: S1 File — (DOCX) [file pntd.0004202.s001.docx]

Supplemental File:

PNTD-D-15-00779: Parasitological, hematological and biochemical characteristics of a model of hyper-microfilariaemic loiasis (*Loa loa*) in the baboon (*Papio anubis*) – Wanji et al.

Legend:

Data and descriptions of those haematological and biochemical parameters whose values that did not vary significantly different from normal.

***Changes in haematological parameters during the course of infection***

**Hemoglobin**

Hemoglobin (Hb) values ranged from 10-16.2g/dl (median: 13.90g/dl) essentially close to normal values (NV) of 9.5-14.5g/dl. Hb values in males ranged from 10-16.2g/dl (median: 14.4g/dl) whilst for females the values were 11-15.2g/dl (median 13.6g/dl); these were significantly different (P>0.01). The Hb values also varied significantly (p>0.05) between different time points and these values showed a moderate increase over time (Fig 3B). BABs 01, 04, 05, 09, 10, 11, 12, 19, had Hb values above the normal range at a number of time points. However, overall the majority of Hb values were within the NV (Fig 3B).

**Red Blood Cells**

RBC counts ranged from 2.65 - 4.3 x10^6^ cells/mm^3^ (median 3.48 x10^6^ cells/mm^3^; NV = 3.76-5.61 x10^6^ cells/mm^3^). Again males differed significantly from females in RBC values (p >0.001) with the values for males being 2.8 - 4.3 x10^6^ (median: 3.7 x 10^6^) cells/mm^3^ whilst in females they ranged from 2.65 - 4.20 (median: 3.39 x 10^6^) cells/mm^3^. RBC values at different time points did not vary significantly (p=0.327) and did not show any significant linear trend (p=0.503). Most RBC values recorded in all animals were below the NV at the different time points (Fig 3A).

**White Blood Cells**

WBC counts varied from 4,000 to 10,200 cells/mm^3^ with a median of 7,000 cells/mm^3^ (NV: 1,800 - 27,400 cells/mm^3^). The figures for males were 4,800-10,200 cells/mm^3^ (Median 7050 cells/mm^3)^ while for females WBC values were 4,000 - 9,000 cells/mm^3^ (Median 7000 cells/mm^3)^. WBC values between males and females did not vary significantly (p=0.524) and the WBC values at different time points showed no significant variation (p=0.570) and no significant linear trend (p=0.969). The total white cell counts were generally all within the NV (Fig 4A).

**Neutrophils**

Absolute neutrophil counts ranged from 1,000-19,500 cells/mm^3^ (Median: 2,250 cells/mm^3^) with the NV given as 576-24660 cells/mm^3^. In males these counts varied from 1,160-19,500 cells/mm^3^ (median 2,384 cells/mm^3^) whilst in females they ranged from 1,000-4,300 cells/mm^3^ (Median 2,200 cells/mm^3^). Absolute neutrophil values between males and females did not vary significantly (p=0.146). The neutrophil counts at different time points did not vary significantly (p=0.084) although there was no linear trend (p=0.756). Neutrophil values were within the NV (Fig 4B).

**Mononuclear Cells**

Absolute mononuclear (lymphocyte + monocyte) counts ranged from 2,400-34,760, cells/mm^3^ with a median of 3,796 cells/mm^3^ (NV: 810-19,728 cells/mm^3^). The values for males were 1,800-39,000 cells/mm^3^ (median: 3,788 cells/mm^3^) while in females these counts ranged from 1,848-6,030 cells/mm^3^ (median: 3,810 cells/mm^3^). These mononuclear cells counts did not vary significantly between males and females (p=0.904). Absolute counts at different time points however did vary significantly (p>0.001) and showed a significant linear trend (p>0.001) Mononuclear counts recorded were within the NV (Fig 4C).

***Changes in biochemical parameters during the course of infection***

**SGPT**

SGPT values ranged from 0-165 IU/L with a median of 22.00 IU/L (NV: 4-84 IU/L). In males SGPT values ranged from 0-165 IU/L; median 23.5 IU/L while in females it ranged from 3-115 IU/L; median 20 IU/L. There was no significant difference between males and females (p=0.342), nor did the SGPT values at different time points vary significantly (p=0.086) or show a significant linear trend (p=0.110). The majority of SGPT values were within the NV, except for BABs 01, 02, 04, 07, 09 and 15 that had values out of the NV at various time points (Fig 6A).

**SGOT**

SGOT values ranged from 0-130 IU/L (NV: 18-69 IU/L) with a median of 30 IU/L. In males SGOT values ranged from 1-130 IU/L (Median = 29 IU/L; normal = 29-69 IU/L) whilst in females it was 0-114 IU/L with a median 30 IU/L (NV: 18-50 IU/L). There was no significant difference in SGPT values between males and females (p=0.342). The SGOT values at different time points varied significantly (p>0.05) although there was no significant linear trend (p=0.356). The majority of SGOT values were within the NV, even though all baboons except BAB 04 showed SGOT values both below or above the NV at different time points (Fig 6B).

**γ-GT**

γ-GT levels ranged from 0-104 IU/L (NV: 11-63 IU/L) with a median of 21.00 IU/L. In males the γ-GT values ranged from 2-104 IU/L (Median 23 IU/L; NV: 23-63 IU/L) while in females it ranged from 0-59 IU/L (Median 20 IU/L; NV: 11-53 IU/L). No significant difference was seen with the SGPT values between males and females (p=0.136). The γ-GT values at different time points varied significantly (p>0.001), and there was a significant linear relationship between microfilaraemia and the duration of infection (p>0.05). BABs 04, 08, 10 and 12 had γ-GT values above the normal range at different time points while BABs 01, 05, 06, 07, 09, 13, 14, and 15 had γ-GT values below the normal range at different time points. Most of the γ-GT values were within the NV (Fig 6C).

**CREATININE**

Creatinine levels ranged from 0-542 mg/L (NV: 5-15 mg/L) with a median of 13.00 mg/L. In males, creatinine values ranged from 1-542 mg/L; median 12 mg/L (NV: 7-15 mg/L) while in females creatinine values ranged from 0-119 mg/L; median 13.5 mg/L (NV: 5-11 mg/L). No significant difference was recorded in creatinine values between males and females (p=0.352). The creatinine values at different time points varied significantly, (p>0.001) although the values did not show any significant linear relationship with the duration of infection (p=0.068). Most of the creatinine values were above the NV at the different time points (Fig 7A).

**GLUCOSE**

Glucose levels ranged from 0.2-2.8 g/L (NV: 0.4-1.36 g/L) with a median of 0.900g/L. In males, glucose values ranged from 0.3-2.6 g/L (Median 0.90 g/L ; NV: 0.65-1.01 g/L) while in females glucose figures were from 0.2-2.8 g/L (Median 0.80 g/L ; NV: 0.4-1.36 g/L). No significant difference was recorded in glucose values between males and females (p=0.141). The glucose values at different time points varied significantly p>0.001), and there was significant linear relationship between blood glucose level and the duration of infection (p>0.001). Majority of the glucose values were within the normal range, although all baboons showed glucose values out of the normal range at different time points (Fig 7B).

**CALCIUM**

Calcium levels ranged from 1-166 mg/L (NV: 74-104 mg/L) with a median of 15.00 mg/L. In males calcium values ranged from 3-106 mg/L (Median 18 mg/L; NV: 82-100 mg/L) while in females it varied from 1-166 mg/L; median 14 mg/L (NV: 74-104 mg/L). No significant difference was recorded in calcium values between males and females (p=0.141). The calcium values varied significantly at different time points (p>0.001) and showed a significant linear relationship with the duration of infection (p>0.001). Majority of the calcium values in all animals at different time points were below the NV (Fig 8A).

**POTASSIUM**

Potassium levels ranged from 0-162 mmol/L (NV: 2.7-5.1 mmol/L) with a median of 6.75 mmol/L. In males potassium values ranged from 0.9-162 mmol/L (median 7.35 mmol/L; normal range: 2.9-5.1 mmol/L) while in females values were 0-85.6 mmol/L; median 6.4 mmol/L (NV: 2.7-5.1 mmol/L). No significant difference was recorded in potassium values between males and females (p=0.462). The potassium values at different time points varied significantly, (p>0.001), and showed a significant negative linear relationship with the duration of infection (p>0.001). The majority of the potassium values in all animals were out of the NV (Fig 8B).
